# Bromidobis[3-(1*H*-pyrazol-1-yl-κ*N* ^2^)propionamide-κ*O*]copper(II) bromide methanol monosolvate

**DOI:** 10.1107/S1600536812038111

**Published:** 2012-09-08

**Authors:** Thomas Wagner, Cristian G. Hrib, Volker Lorenz, Frank T. Edelmann, John W. Gilje

**Affiliations:** a Chemisches Institut, Otto-von-Guericke-Universität Magdeburg, Universitätsplatz 2, 39106 Magdeburg, Germany; b Department of Chemistry and Biochemistry, James Madison University, Harrisonburg, VA 22807, USA

## Abstract

The title copper(II) *N*-pyrazolylpropanamide (PPA) complex, [CuBr(PPA)_2_]Br, was obtained in 78% yield by treatment of CuBr_2_ with an excess of the ligand in methanol. Crystallization from the mother liquid afforded the title compound, *i.e.* the methanol solvate [CuBr(C_6_H_9_N_3_O)_2_]Br·CH_3_OH or [CuBr(PPA)_2_]Br·MeOH, as bright green crystals. In the solid state, the title salt comprises isolated [CuBr(PPA)_2_]^+^ cations, separated bromide ions and methanol of crystallization. In the cation, the central Cu^II^ ion is coordinated by two *N*,*O*-chelating PPA ligands and one Br^−^ ion. The coordination geometry around the Cu^II^ ion is distorted trigonal–bipyramidal with the bromide ligand and the amide O atoms occupying the equatorial positions [Cu—Br = 2.4443 (4) Å; Cu—O = 2.035 (2) and 2.179 (2) Å], while the pyrazole N atoms coordinate in the axial positions [Cu—N = 1.975 (2) and 1.976 (2) Å]. In the crystal, the three constituents are linked by N—H⋯Br, O—H⋯Br, and N—H⋯O hydrogen bonds, forming a three-dimensional network.

## Related literature

For related complexes containing multifunctional ligands with substituted pyrazole groups, see: Gracia-Anton *et al.* (2003 ▶); Mukherjee (2000 ▶); Pal *et al.* (2005 ▶); Shaw *et al.* (2004 ▶). For acryl­amide complexes, see: Girma *et al.* (2005*a* ▶,*b* ▶,*c* ▶, 2006 ▶). For related complexes containing 3-pyrazol-1-yl-propionamide, see: Girma *et al.* (2008 ▶); Wagner *et al.* (2012 ▶).
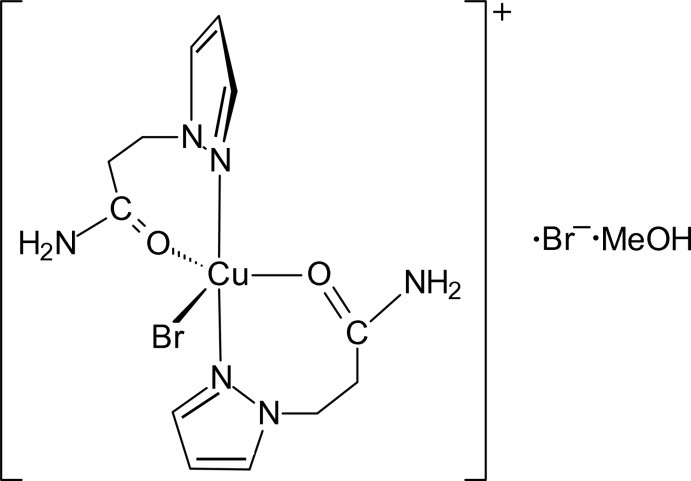


## Experimental

### 

#### Crystal data

[CuBr(C_6_H_9_N_3_O)_2_]Br·CH_4_O
*M* *_r_* = 533.73
Monoclinic, 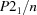

*a* = 10.5075 (4) Å
*b* = 12.6951 (4) Å
*c* = 15.1551 (7) Å
β = 102.821 (3)°
*V* = 1971.19 (13) Å^3^
*Z* = 4
Mo *K*α radiation
μ = 5.19 mm^−1^
*T* = 150 K
0.40 × 0.40 × 0.30 mm

#### Data collection

Stoe IPDS 2T diffractometer
Absorption correction: multi-scan (Blessing, 1995 ▶) *T*_min_ = 0.046, *T*_max_ = 0.139
22729 measured reflections
5320 independent reflections
4728 reflections with *I* > 2σ(*I*)
*R*_int_ = 0.048

#### Refinement

*R*[*F* ^2^ > 2σ(*F* ^2^)] = 0.041
*wR*(*F* ^2^) = 0.079
*S* = 1.21
5320 reflections
231 parameters
H atoms treated by a mixture of independent and constrained refinement
Δρ_max_ = 0.74 e Å^−3^
Δρ_min_ = −0.70 e Å^−3^

### 

Data collection: *X-AREA* (Stoe & Cie, 2002 ▶); cell refinement: *X-AREA*; data reduction: *X-RED* (Stoe & Cie, 2002 ▶); program(s) used to solve structure: *SHELXS97* (Sheldrick, 2008 ▶); program(s) used to refine structure: *SHELXL97* (Sheldrick, 2008 ▶); molecular graphics: *XP* in *SHELXTL* (Sheldrick, 2008 ▶); software used to prepare material for publication: *SHELXL97*.

## Supplementary Material

Crystal structure: contains datablock(s) I, global. DOI: 10.1107/S1600536812038111/qk2039sup1.cif

Structure factors: contains datablock(s) I. DOI: 10.1107/S1600536812038111/qk2039Isup2.hkl

Additional supplementary materials:  crystallographic information; 3D view; checkCIF report

## Figures and Tables

**Table 1:** Hydrogen-bond geometry (Å, °)

*D*—H⋯*A*	*D*—H	H⋯*A*	*D*⋯*A*	*D*—H⋯*A*
N1—H1*NB*⋯O3^i^	0.88	2.07	2.926 (3)	163
N1—H1*NA*⋯Br2	0.88	2.56	3.434 (3)	173
N4—H4*NA*⋯O3^ii^	0.88	2.09	2.956 (3)	168
O3—H1*O*⋯Br2	0.81 (5)	2.41 (5)	3.215 (3)	175 (5)
N4—H4*NB*⋯Br2^iii^	0.88	2.71	3.548 (3)	160

Symmetry codes: (i) 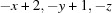; (ii) 
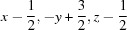
; (iii) 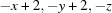.

**Fig. 1.**
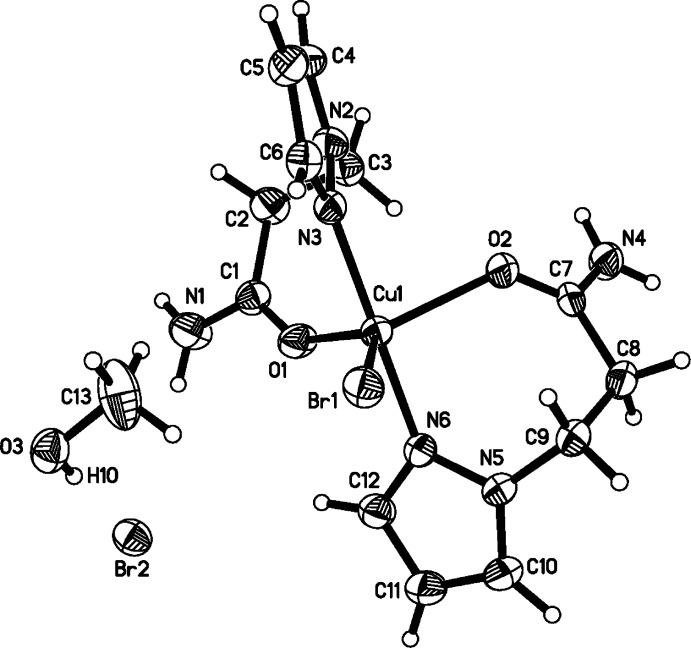
The ion-pair of the title compound in the crystal. Thermal ellipsoids represent 50% probability levels. H-atom radii are arbitrary.

**Fig. 2.**
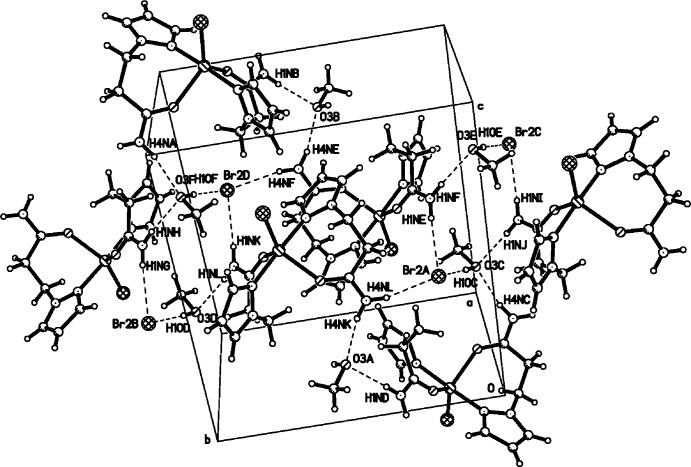
Packing diagram of the title compound. Hydrogen bonds: 1 N(1)—H(1NB)···O(3)i, 2 N(1)—H(1NA)···Br(2), 3 N(4)—H(4NA)···O(3)ii, 4 O(3)—H(1O)···Br(2), 5 N(4)—H(4NB)···Br(2)iii. See Table 1 for details.

## supplementary crystallographic information

### Comment

Currently there is a considerable interest in the use of multifunctional ligands containing substituted pyrazole groups because of their potential applications in catalysis and their ability to form complexes that mimic structural and catalytic functions in metalloproteins (Gracia-Anton *et al.,* 2003; Mukherjee, 2000; Pal *et al.,* 2005; Shaw *et al.*, 2004). As part of our continuing interest in the coordination chemistry of acrylamide (Girma *et al.,* 2005*a*; Girma *et al.,* 2005*b*; Girma *et al.,* 2005*c*; Girma *et al.,* 2006) and acrylamide-based ligands we have earlier synthesized and characterized an acrylamide-derived pyrazole ligand, *N*-pyrazolylpropanamide (= PPA) (Girma *et al.*, 2008). This ligand is readily accessible in large quantities by base-catalyzed Michael addition of pyrazole to acrylamide. The first PPA complexes to be reported were (PPA)_2_CuCl_2_ and (PPA)_4_Co_3_Cl_6_ with copper(II) and cobalt(II) chlorides, respectively. Both complexes contain the ligand in a seven-membered ring *N,O*-chelating fashion. Especially remarkable was the unusual zwitterionic structure of the trinuclear cobalt complex, which additionally comprises bridging *N*-pyrazolylpropanamide ligands (Girma *et al.,* 2008). Most recently, the syntheses and single-crystal X-ray structures of several new first-row transition metal complexes containing the multifunctional acrylamide-derived PPA have been reported. The general synthesis involved treatment of the appropriate transition metal salts with an excess of PPA in ethanolic solution in the presence of triethylorthoformate as dehydrating agent. This way the perchlorates of iron(II) and cobalt(II) afforded the complexes [(PPA)_2_M(EtOH)_2_](ClO_4_)_2_ (*M* = Fe, Co) in good yields (82% resp. 85%). Light green (PPA)_2_NiCl_2_ has been obtained analogously from NiCl_2_.6H_2_O. Hydration of (PPA)_2_NiCl_2_ afforded the dark green cationic nickel(II) complex [(PPA)_2_Ni(H_2_O)_4_]Cl_2_. In the complexes with *M* = Fe, Co, and Ni the *N*-pyrazolylpropanamide acts as *N,O*-chelating ligand. In contrast, monodentate *N*-coordination *via* the pyrazolyl ring was found for the dicoordinate silver(I) complex [(PPA)_2_Ag]NO_3_.H_2_O (Wagner *et al.,* 2012).

In the course of these investigations we now studied the reaction of copper(II) dibromide with PPA. A reaction carried out in methanol solution using an excess over 2 equivalents of PPA afforded a green, microcrystalline solid which was shown by elemental analysis and its IR spectrum to be the bromide analogue of the previously reported (PPA)_2_CuCl_2_ (Girma *et al.*, 2008), *i.e.* (PPA)_2_CuBr_2_. Comparison of the IR spectra of the free ligand PPA and (PPA)_2_CuBr_2_ revealed a significant decrease to lower wavenumbers in the CO absorptions (1690 cm^-1^ in PPA *vs.* 1661 cm^-1^ in (PPA)_2_CuBr_2_ and 1664 cm^-1^ in (PPA)_2_CuCl_2_). This CO absorption shift is consistent with a decrease in the electron density of the amide carbonyl moiety resulting from coordination to the cationic copper(II) center. Crystallization of (PPA)_2_CuBr_2_ directly from the mother liquid afforded the title compound, *i.e.* the methanol-solvate, as bright green, X-ray quality single crystals. Surprisingly, the X-ray crystal structure determination revealed the presence of the ionic product [CuBr(PPA)_2_]Br.MeOH with 5-coordinate copper. In the solid state, [CuBr(PPA)_2_]Br.MeOH comprises isolated [CuBr(PPA)_2_]^+^ cations, separated bromide ions and methanol of crystallization (Fig. 1). In the cation, the central Cu^2+^ ion is coordinated by two *N,O*-chelating PPA ligands and one Br^-^ ion. The coordination geometry around copper can be best described as distorted trigonal-bipyramidal with the bromo ligand and the amide O atoms occupying the equatorial positions (Cu—Br 2.4443 (4) Å; Cu—O 2.035 (2) and 2.179 (2) Å), while the pyrazolyl N-atoms coordinate in the axial positions (Cu—N 1.975 (2) and 1.976 (2) Å). This is in contrast to the neutral hexacoordinate chloro-analogue (PPA)_2_CuCl_2_ in which the coordination geometry around the central Cu^2+^ ion is octahedral (Girma *et al.*, 2008). The angles at Cu in the equatorial plane of the [CuBr(PPA)_2_]^+^ cation are 109.30 (8)° (O1—Cu—O2), 116.80 (6)° (O2—Cu—Br1), and 133.76 (6)° (O1—Cu—Br1), respectively. The axial angle at Cu (N3—Cu—N6) is 174.53 (9)°. The planes of the opposing pyrazole rings are inclined to each other by ~71°.

Like all previously reported transition metal PPA complexes (Wagner *et al.*, 2012), the title compound also forms a hydrogen-bonded network in the solid state (Fig. 2). The crystal structure consists of chains of [CuBr(PPA)_2_]^+^ cations linked by N—H···Br, O—H···Br, and N—H···O hydrogen bonds. The uncoordinated bromide ions are connected *via* O—H···Br hydrogen bonds to the methanol of crystallization and *via* two N—H···Br interactions with the amide NH_2_ groups of different cations. N—H···O hydrogen bonds between amide NH_2_ groups and the methanol of crystallization interconnect the chains.

### Experimental

A solution of CuBr_2_ (0.6 g, 1.69 mmol) in methanol (50 ml) was combined with a solution of *N*-pyrazolylpropanamide (= PPA, 0.8 g, 5.5 mmol) in methanol (30 ml). After stirring for 24 h at room temperature, the reaction mixture was concentrated *in vacuo* to a total volume of *ca* 50 ml, whereupon a large quantity of the title compound precipitated in its unsolvated form [CuBr(PPA)_2_]Br in the form of a green, microcrystalline solid in 78% yield (1.05 g). Bright green, X-ray quality crystals of the title compound [CuBr(PPA)_2_]Br.MeOH were obtained upon standing of the mother liquid at room temperature for 14 d.

Anal. Calcd. for unsolvated C_12_H_18_Br_2_CuN_6_O_2_ (*M*_r_ = 501.66): C 28.73%; H 3.62%; N 16.75%; Br 31.86%. Found: C 28.35%; H 3.46%; N 17.03%; Br 31.47%. IR (KBr): 3436*m ν*(N—H), 3336*m ν*(N—H), 3298*m*, 3188*m*, 3020*w*, 2964*m*, 2847*w*, 1661*s ν*(C=O), 1594*m; ν*(C=C), 1494*w*, 1450*m*, 1429*m*, 1418*m*, 1375*w*, 1325*m*, 1285*m*, 1262*s*, 1230*m*, 1175*m*, 1098*vs*, 1022*vs*, 957*m*, 949*m*, 868*w*, 801*vs*, 768*m*, 759*m*, 628*m* cm^-1^.

### Refinement

The hydrogen atoms were included in idealized positions using a riding model, with N—H = 0.88 Å, aromatic C—H = 0.95 Å, methylene C—H = 0.99 Å [*U*_iso_(H) = 1.2*U*_eq_(C)] and methyl C—H = 0.98 Å [*U*_iso_(H) = 1.5*U*_eq_(C)]. The O—H proton of methanol was freely refined.

### Crystal data

**Table:**

[CuBr(C_6_H_9_N_3_O)_2_]Br·CH_4_O	*F*(000) = 1060
*M**_r_* = 533.73	*D*_x_ = 1.798 Mg m^−^^3^
Monoclinic, *P*2_1_/*n*	Mo *K*α radiation, λ = 0.71073 Å
*a* = 10.5075 (4) Å	Cell parameters from 30970 reflections
*b* = 12.6951 (4) Å	θ = 2.0–29.6°
*c* = 15.1551 (7) Å	µ = 5.19 mm^−^^1^
β = 102.821 (3)°	*T* = 150 K
*V* = 1971.19 (13) Å^3^	Prism, red
*Z* = 4	0.40 × 0.40 × 0.30 mm

### Data collection

**Table:**

Stoe IPDS 2T diffractometer	5320 independent reflections
Radiation source: fine-focus sealed tube	4728 reflections with *I* > 2σ(*I*)
Graphite monochromator	*R*_int_ = 0.048
Detector resolution: 6.67 pixels mm^-1^	θ_max_ = 29.2°, θ_min_ = 2.1°
ω and φ scans	*h* = −14→14
Absorption correction: multi-scan (Blessing, 1995)	*k* = −16→17
*T*_min_ = 0.046, *T*_max_ = 0.139	*l* = −20→20
22729 measured reflections	

### Refinement

**Table:**

Refinement on *F*^2^	Primary atom site location: structure-invariant direct methods
Least-squares matrix: full	Secondary atom site location: difference Fourier map
*R*[*F*^2^ > 2σ(*F*^2^)] = 0.041	Hydrogen site location: inferred from neighbouring sites
*wR*(*F*^2^) = 0.079	H atoms treated by a mixture of independent and constrained refinement
*S* = 1.21	*w* = 1/[σ^2^(*F*_o_^2^) + (0.0324*P*)^2^ + 1.2084*P*] where *P* = (*F*_o_^2^ + 2*F*_c_^2^)/3
5320 reflections	(Δ/σ)_max_ = 0.001
231 parameters	Δρ_max_ = 0.74 e Å^−^^3^
0 restraints	Δρ_min_ = −0.70 e Å^−^^3^

### Special details

**Table:**

Geometry. All e.s.d.'s (except the e.s.d. in the dihedral angle between two l.s. planes) are estimated using the full covariance matrix. The cell e.s.d.'s are taken into account individually in the estimation of e.s.d.'s in distances, angles and torsion angles; correlations between e.s.d.'s in cell parameters are only used when they are defined by crystal symmetry. An approximate (isotropic) treatment of cell e.s.d.'s is used for estimating e.s.d.'s involving l.s. planes.
Refinement. Refinement of *F*^2^ against ALL reflections. The weighted *R*-factor *wR* and goodness of fit *S* are based on *F*^2^, conventional *R*-factors *R* are based on *F*, with *F* set to zero for negative *F*^2^. The threshold expression of *F*^2^ > σ(*F*^2^) is used only for calculating *R*-factors(gt) *etc*. and is not relevant to the choice of reflections for refinement. *R*-factors based on *F*^2^ are statistically about twice as large as those based on *F*, and *R*- factors based on ALL data will be even larger.

### Fractional atomic coordinates and isotropic or equivalent isotropic displacement parameters (Å^2^)

**Table:**

	*x*	*y*	*z*	*U*_iso_*/*U*_eq_	
C1	0.8900 (3)	0.6996 (2)	−0.06693 (18)	0.0305 (5)	
C2	0.7834 (3)	0.6821 (3)	−0.15080 (18)	0.0370 (6)	
H2A	0.8183	0.6990	−0.2047	0.044*	
H2B	0.7594	0.6065	−0.1543	0.044*	
C3	0.6616 (3)	0.7466 (2)	−0.15415 (18)	0.0336 (6)	
H3A	0.6871	0.8199	−0.1361	0.040*	
H3B	0.6096	0.7481	−0.2172	0.040*	
C4	0.4774 (3)	0.6412 (3)	−0.1195 (2)	0.0391 (7)	
H4	0.4395	0.6181	−0.1792	0.047*	
C5	0.4364 (3)	0.6148 (2)	−0.0431 (2)	0.0397 (7)	
H5	0.3648	0.5710	−0.0386	0.048*	
C6	0.5219 (3)	0.6655 (2)	0.02672 (19)	0.0319 (5)	
H6	0.5181	0.6613	0.0887	0.038*	
C7	0.6471 (2)	1.0403 (2)	−0.04321 (17)	0.0261 (5)	
C8	0.6850 (3)	1.1169 (2)	0.03418 (19)	0.0338 (6)	
H8A	0.6095	1.1633	0.0348	0.041*	
H8B	0.7567	1.1619	0.0229	0.041*	
C9	0.7282 (3)	1.0670 (2)	0.12653 (17)	0.0294 (5)	
H9A	0.7269	1.1211	0.1734	0.035*	
H9B	0.6652	1.0113	0.1333	0.035*	
C10	0.9713 (3)	1.0667 (2)	0.1824 (2)	0.0355 (6)	
H10	0.9809	1.1329	0.2126	0.043*	
C11	1.0701 (3)	1.0012 (3)	0.1729 (2)	0.0386 (7)	
H11	1.1611	1.0120	0.1948	0.046*	
C12	1.0095 (3)	0.9150 (2)	0.12412 (19)	0.0330 (6)	
H12	1.0537	0.8558	0.1069	0.040*	
C13	0.9020 (4)	0.5691 (3)	0.1716 (3)	0.0615 (11)	
H13A	0.8351	0.5250	0.1892	0.092*	
H13B	0.8811	0.5784	0.1058	0.092*	
H13C	0.9044	0.6380	0.2011	0.092*	
N1	0.9979 (3)	0.6464 (2)	−0.06237 (19)	0.0463 (7)	
H1NA	1.0636	0.6541	−0.0154	0.056*	
H1NB	1.0049	0.6030	−0.1063	0.056*	
N2	0.5811 (2)	0.70573 (19)	−0.09558 (15)	0.0310 (5)	
N3	0.6103 (2)	0.72132 (17)	−0.00452 (14)	0.0268 (4)	
N4	0.6262 (2)	1.0830 (2)	−0.12477 (15)	0.0340 (5)	
H4NA	0.6005	1.0434	−0.1731	0.041*	
H4NB	0.6379	1.1511	−0.1307	0.041*	
N5	0.8581 (2)	1.02134 (18)	0.14175 (14)	0.0279 (4)	
N6	0.8801 (2)	0.92774 (17)	0.10504 (14)	0.0269 (4)	
O1	0.8780 (2)	0.76128 (17)	−0.00574 (13)	0.0347 (4)	
O2	0.63174 (19)	0.94471 (15)	−0.03194 (13)	0.0311 (4)	
O3	1.0247 (2)	0.51986 (18)	0.19866 (14)	0.0375 (5)	
Br1	0.69094 (3)	0.80080 (2)	0.204039 (17)	0.03143 (7)	
Br2	1.23867 (3)	0.66180 (2)	0.131810 (19)	0.03514 (8)	
Cu1	0.74194 (3)	0.82447 (2)	0.05558 (2)	0.02424 (8)	
H1O	1.078 (5)	0.559 (4)	0.184 (3)	0.065 (14)*	

### Atomic displacement parameters (Å^2^)

**Table:**

	*U*^11^	*U*^22^	*U*^33^	*U*^12^	*U*^13^	*U*^23^
C1	0.0342 (13)	0.0303 (13)	0.0272 (12)	0.0011 (11)	0.0074 (10)	−0.0035 (10)
C2	0.0414 (15)	0.0441 (17)	0.0247 (12)	0.0063 (13)	0.0054 (11)	−0.0076 (11)
C3	0.0390 (15)	0.0367 (15)	0.0235 (11)	0.0061 (12)	0.0033 (10)	−0.0008 (10)
C4	0.0333 (14)	0.0382 (15)	0.0401 (15)	0.0011 (12)	−0.0040 (12)	−0.0116 (12)
C5	0.0337 (14)	0.0304 (14)	0.0536 (18)	−0.0014 (12)	0.0066 (13)	−0.0055 (13)
C6	0.0340 (13)	0.0264 (13)	0.0349 (13)	0.0019 (11)	0.0066 (11)	0.0021 (10)
C7	0.0242 (11)	0.0241 (11)	0.0289 (11)	0.0065 (9)	0.0034 (9)	0.0009 (9)
C8	0.0427 (15)	0.0227 (12)	0.0331 (13)	0.0043 (11)	0.0020 (11)	−0.0025 (10)
C9	0.0324 (13)	0.0264 (12)	0.0293 (12)	0.0019 (10)	0.0064 (10)	−0.0043 (10)
C10	0.0378 (14)	0.0306 (14)	0.0350 (14)	−0.0056 (12)	0.0011 (11)	−0.0061 (11)
C11	0.0291 (14)	0.0406 (16)	0.0427 (15)	−0.0041 (12)	0.0008 (12)	−0.0059 (13)
C12	0.0283 (13)	0.0360 (14)	0.0331 (13)	0.0015 (11)	0.0038 (10)	−0.0034 (11)
C13	0.065 (2)	0.058 (2)	0.073 (3)	0.027 (2)	0.039 (2)	0.025 (2)
N1	0.0405 (14)	0.0560 (17)	0.0387 (13)	0.0153 (13)	0.0012 (11)	−0.0196 (12)
N2	0.0329 (11)	0.0318 (12)	0.0247 (10)	0.0035 (9)	−0.0010 (8)	−0.0022 (9)
N3	0.0312 (11)	0.0234 (10)	0.0237 (9)	0.0026 (8)	0.0013 (8)	−0.0005 (8)
N4	0.0432 (13)	0.0277 (11)	0.0292 (11)	0.0018 (10)	0.0040 (10)	0.0035 (9)
N5	0.0307 (11)	0.0259 (11)	0.0259 (10)	−0.0003 (9)	0.0034 (8)	−0.0050 (8)
N6	0.0276 (10)	0.0257 (10)	0.0267 (10)	0.0028 (8)	0.0045 (8)	−0.0039 (8)
O1	0.0329 (10)	0.0386 (11)	0.0322 (9)	0.0020 (9)	0.0063 (8)	−0.0129 (8)
O2	0.0359 (10)	0.0240 (9)	0.0295 (9)	0.0021 (8)	−0.0008 (7)	0.0014 (7)
O3	0.0475 (13)	0.0313 (11)	0.0350 (10)	0.0007 (9)	0.0118 (9)	0.0026 (8)
Br1	0.03704 (14)	0.03321 (14)	0.02379 (11)	0.00164 (11)	0.00622 (9)	0.00264 (9)
Br2	0.03229 (14)	0.03990 (16)	0.03307 (14)	−0.00045 (11)	0.00691 (10)	0.00324 (11)
Cu1	0.02684 (15)	0.02305 (14)	0.02175 (14)	0.00120 (12)	0.00305 (11)	−0.00200 (10)

### Geometric parameters (Å, º)

**Table:**

C1—O1	1.241 (3)	C9—H9B	0.9900
C1—N1	1.309 (4)	C10—N5	1.343 (3)
C1—C2	1.513 (4)	C10—C11	1.363 (5)
C2—C3	1.510 (4)	C10—H10	0.9500
C2—H2A	0.9900	C11—C12	1.393 (4)
C2—H2B	0.9900	C11—H11	0.9500
C3—N2	1.451 (4)	C12—N6	1.336 (3)
C3—H3A	0.9900	C12—H12	0.9500
C3—H3B	0.9900	C13—O3	1.410 (4)
C4—N2	1.347 (4)	C13—H13A	0.9800
C4—C5	1.365 (5)	C13—H13B	0.9800
C4—H4	0.9500	C13—H13C	0.9800
C5—C6	1.385 (4)	N1—H1NA	0.8800
C5—H5	0.9500	N1—H1NB	0.8800
C6—N3	1.336 (4)	N2—N3	1.360 (3)
C6—H6	0.9500	N3—Cu1	1.975 (2)
C7—O2	1.241 (3)	N4—H4NA	0.8800
C7—N4	1.323 (3)	N4—H4NB	0.8800
C7—C8	1.507 (4)	N5—N6	1.354 (3)
C8—C9	1.511 (4)	N6—Cu1	1.976 (2)
C8—H8A	0.9900	O1—Cu1	2.035 (2)
C8—H8B	0.9900	O2—Cu1	2.179 (2)
C9—N5	1.453 (3)	O3—H1O	0.81 (5)
C9—H9A	0.9900	Br1—Cu1	2.4443 (4)
			
O1—C1—N1	121.1 (3)	C10—C11—H11	127.3
O1—C1—C2	122.8 (3)	C12—C11—H11	127.3
N1—C1—C2	116.1 (2)	N6—C12—C11	110.1 (3)
C3—C2—C1	114.3 (2)	N6—C12—H12	124.9
C3—C2—H2A	108.7	C11—C12—H12	124.9
C1—C2—H2A	108.7	O3—C13—H13A	109.5
C3—C2—H2B	108.7	O3—C13—H13B	109.5
C1—C2—H2B	108.7	H13A—C13—H13B	109.5
H2A—C2—H2B	107.6	O3—C13—H13C	109.5
N2—C3—C2	113.0 (2)	H13A—C13—H13C	109.5
N2—C3—H3A	109.0	H13B—C13—H13C	109.5
C2—C3—H3A	109.0	C1—N1—H1NA	120.0
N2—C3—H3B	109.0	C1—N1—H1NB	120.0
C2—C3—H3B	109.0	H1NA—N1—H1NB	120.0
H3A—C3—H3B	107.8	C4—N2—N3	110.4 (2)
N2—C4—C5	108.1 (3)	C4—N2—C3	126.8 (2)
N2—C4—H4	126.0	N3—N2—C3	122.5 (2)
C5—C4—H4	126.0	C6—N3—N2	105.3 (2)
C4—C5—C6	105.1 (3)	C6—N3—Cu1	131.25 (19)
C4—C5—H5	127.5	N2—N3—Cu1	122.79 (18)
C6—C5—H5	127.5	C7—N4—H4NA	120.0
N3—C6—C5	111.1 (3)	C7—N4—H4NB	120.0
N3—C6—H6	124.4	H4NA—N4—H4NB	120.0
C5—C6—H6	124.4	C10—N5—N6	110.5 (2)
O2—C7—N4	122.0 (2)	C10—N5—C9	127.4 (2)
O2—C7—C8	122.9 (2)	N6—N5—C9	121.7 (2)
N4—C7—C8	115.0 (2)	C12—N6—N5	106.0 (2)
C7—C8—C9	115.0 (2)	C12—N6—Cu1	128.97 (19)
C7—C8—H8A	108.5	N5—N6—Cu1	124.08 (17)
C9—C8—H8A	108.5	C1—O1—Cu1	141.98 (19)
C7—C8—H8B	108.5	C7—O2—Cu1	134.49 (17)
C9—C8—H8B	108.5	C13—O3—H1O	107 (3)
H8A—C8—H8B	107.5	N3—Cu1—N6	174.53 (9)
N5—C9—C8	113.1 (2)	N3—Cu1—O1	91.11 (9)
N5—C9—H9A	109.0	N6—Cu1—O1	84.46 (9)
C8—C9—H9A	109.0	N3—Cu1—O2	87.74 (8)
N5—C9—H9B	109.0	N6—Cu1—O2	90.72 (8)
C8—C9—H9B	109.0	O1—Cu1—O2	109.30 (8)
H9A—C9—H9B	107.8	N3—Cu1—Br1	93.94 (7)
N5—C10—C11	107.9 (3)	N6—Cu1—Br1	91.43 (7)
N5—C10—H10	126.0	O1—Cu1—Br1	133.76 (6)
C11—C10—H10	126.0	O2—Cu1—Br1	116.80 (6)
C10—C11—C12	105.4 (3)		
			
O1—C1—C2—C3	0.5 (4)	C9—N5—N6—C12	173.5 (2)
N1—C1—C2—C3	−178.8 (3)	C10—N5—N6—Cu1	170.06 (19)
C1—C2—C3—N2	−76.3 (3)	C9—N5—N6—Cu1	−16.9 (3)
N2—C4—C5—C6	−0.8 (3)	N1—C1—O1—Cu1	−150.9 (3)
C4—C5—C6—N3	0.6 (3)	C2—C1—O1—Cu1	29.7 (5)
O2—C7—C8—C9	11.3 (4)	N4—C7—O2—Cu1	136.7 (2)
N4—C7—C8—C9	−171.4 (3)	C8—C7—O2—Cu1	−46.1 (4)
C7—C8—C9—N5	74.4 (3)	C6—N3—Cu1—O1	137.5 (2)
N5—C10—C11—C12	0.3 (4)	N2—N3—Cu1—O1	−52.8 (2)
C10—C11—C12—N6	0.0 (4)	C6—N3—Cu1—O2	−113.2 (2)
C5—C4—N2—N3	0.7 (3)	N2—N3—Cu1—O2	56.5 (2)
C5—C4—N2—C3	175.0 (3)	C6—N3—Cu1—Br1	3.5 (2)
C2—C3—N2—C4	−95.8 (3)	N2—N3—Cu1—Br1	173.20 (19)
C2—C3—N2—N3	77.8 (3)	C12—N6—Cu1—O1	−29.6 (2)
C5—C6—N3—N2	−0.2 (3)	N5—N6—Cu1—O1	163.2 (2)
C5—C6—N3—Cu1	170.8 (2)	C12—N6—Cu1—O2	−139.0 (2)
C4—N2—N3—C6	−0.4 (3)	N5—N6—Cu1—O2	53.9 (2)
C3—N2—N3—C6	−174.9 (2)	C12—N6—Cu1—Br1	104.2 (2)
C4—N2—N3—Cu1	−172.32 (19)	N5—N6—Cu1—Br1	−62.93 (19)
C3—N2—N3—Cu1	13.2 (3)	C1—O1—Cu1—N3	12.4 (3)
C11—C10—N5—N6	−0.4 (3)	C1—O1—Cu1—N6	−164.4 (3)
C11—C10—N5—C9	−173.0 (3)	C1—O1—Cu1—O2	−75.5 (3)
C8—C9—N5—C10	94.8 (3)	C1—O1—Cu1—Br1	109.0 (3)
C8—C9—N5—N6	−77.1 (3)	C7—O2—Cu1—N3	−177.4 (3)
C11—C12—N6—N5	−0.3 (3)	C7—O2—Cu1—N6	−2.6 (3)
C11—C12—N6—Cu1	−169.2 (2)	C7—O2—Cu1—O1	−87.0 (3)
C10—N5—N6—C12	0.4 (3)	C7—O2—Cu1—Br1	89.4 (3)

### Hydrogen-bond geometry (Å, º)

**Table:**

*D*—H···*A*	*D*—H	H···*A*	*D*···*A*	*D*—H···*A*
N1—H1*NB*···O3^i^	0.88	2.07	2.926 (3)	163
N1—H1*NA*···Br2	0.88	2.56	3.434 (3)	173
N4—H4*NA*···O3^ii^	0.88	2.09	2.956 (3)	168
O3—H1*O*···Br2	0.81 (5)	2.41 (5)	3.215 (3)	175 (5)
N4—H4*NB*···Br2^iii^	0.88	2.71	3.548 (3)	160

Symmetry codes: (i) −*x*+2, −*y*+1, −*z*; (ii) *x*−1/2, −*y*+3/2, *z*−1/2; (iii) −*x*+2, −*y*+2, −*z*.
